# Supplementary material for: Microencapsulation of Chilean Papaya Waste Extract and Its Impact on Physicochemical and Bioactive Properties
Source: Antioxidants (Basel). 2023 Oct 23;12(10):1900. doi: 10.3390/antiox12101900 (PMC10604294; doi:10.3390/antiox12101900)
Supplement: Supplementary file 1 [file antioxidants-12-01900-s001.zip › antioxidants-2668189-supplementary.pdf]

antioxidants

MDPI

Article

Microencapsulation of Chilean Papaya Waste Extract and Its Impact on Physicochemical and Bioactive Properties

Yihajara Fuentes[1], Claudia Giovagnoli-Vicuña[1*], Mario Faundez[2] and Ady Giordano[1*]

1 Departamento de Química Inorgánica, Escuela de Química, Facultad de Química y de Farmacia, Pontificia Universidad Católica de Chile, Macul, Chile.; yyfuentes@uc.cl
2 Departamento de Farmacia, Escuela de Química y Farmacia, Facultad de Química y de Farmacia, Pontificia Universidad Católica de Chile, Macul, Chile; mfaundeza@uc.cl
* Correspondence: agiordano@uc.cl; ccgiovagnoli@uc.cl

Table S1. Parameters for UHPLC-MS/MS determination of polyphenols.

Polyphenol	Retention time (min)	Product Ion 1 (m/z)	Product Ion 2 (*m/z*)	Collision Energy (V)
**Gallic Acid**	3.53	124.9	78.9	-70
**Chlorogenic Acid**	6.99	191	85	-75
**Caffeic Acid**	7.2	135	133.9	-70
**Coumaric Acid**	8.53	119	92.8	-70
**Rutin**	9.63	299.8	300.5	-70
**Quercetin**	11.74	150.9	178.8	-70
**Ferulic Acid**	11.76	146.9	102.9	-5
